# Breaking Down Linear Low-Density Polyethylene (LLDPE) Using Fungal Mycelium (Part A): A Path Towards Sustainable Waste Management and Its Possible Economic Impacts

**DOI:** 10.3390/life15050755

**Published:** 2025-05-08

**Authors:** Worawoot Aiduang, Kritsana Jatuwong, Kingkarn Ratanapong, Thanaporn Promjaidee, Orlavanh Xayyavong, Sinang Hongsanan, Saisamorn Lumyong

**Affiliations:** 1 Office of Research Administration, Chiang Mai University, Chiang Mai 50200, Thailand; worawoot.aiduang@cmu.ac.th (W.A.); kritsana.ja@cmu.ac.th (K.J.); 2 Department of Biology, Faculty of Science, Chiang Mai University, Chiang Mai 50200, Thailand; xorlavanh@yahoo.com; 3 Yupparaj Wittayalai School, Chiang Mai 50200, Thailand; kingkarn8090@gmail.com (K.R.); thanapornpoona@gmail.com (T.P.); 4 Shenzhen Key Laboratory of Microbial Genetic Engineering, College of Life Science and Oceanography, Shenzhen University, Shenzhen 518060, China; 5 Center of Excellence in Microbial Diversity and Sustainable Utilization, Chiang Mai University, Chiang Mai 50200, Thailand; 6 Academy of Science, The Royal Society of Thailand, Bangkok 10300, Thailand

**Keywords:** fungal bioremediation, LLDPE plastic, mycelium application, sustainable waste management, SDG 11 and 15

## Abstract

Linear low-density polyethylene (LLDPE) waste presents a major environmental concern due to its high and widespread use. This study explores the potential of fungal mycelium as a bioremediation solution for LLDPE degradation, by evaluating on mycelial growth efficiency, ligninolytic enzyme activity, weight loss, surface morphology changes, and economic feasibility. Among the tested fungal species, *Schizophyllum commune* WE032, *Lentinus sajor-caju* TBRC6266, and *Trametes flavida* AM011, *S. commune* demonstrated the most vigorous mycelial expansion (20.53 mm/day) and highest biomass accumulation (276.87 mg). Screening for ligninolytic enzymes revealed significant laccase (Lac) and manganese peroxidase (MnP) activity in all three species indicating their potential in polymer degradation. Weight loss analysis showed that *S. commune* achieved the greatest LLDPE degradation (1.182% after 30 days), highlighting its enzymatic and metabolic efficiency in breaking down synthetic polymers. Surface morphology studies supported these findings, revealing substantial erosion was observed in LLDPE sheets treated with *S. commune* and *L. sajor-caju*, confirming their effectiveness in polymer disruption. FTIR analysis indicated the formation of new functional groups and alterations in the carbon backbone, suggesting active depolymerization processes. Economic evaluation demonstrated that fungal biodegradation is a cost-effective and environmentally sustainable strategy, aligning with circular economy principles by enabling the generation of value-added products from plastic waste. Additionally, fungal-based waste treatment aligns with circular economy principles, generating value-added products while mitigating plastic pollution. These findings highlight fungal mycelium’s potential for plastic waste management, advocating for further research on optimizing growth conditions, enhancing enzyme expression, and scaling industrial applications. Future research will focus on integrating fungal bioremediation with biomass residues from agricultural and forestry sectors, offering a comprehensive solution for waste management and environmental sustainability.

## 1. Introduction

The accumulation of plastic waste in the global ecosystem poses a significant environmental challenge, particularly from low-density and linear low-density polyethylene (LDPE and LLDPE). These widely used thermoplastics are prevalent in packaging, construction, automotive, electronics, agriculture, and other consumer goods, contributing significantly to plastic pollution due to their durability and resistance to degradation [1,2]. The inherent resistance of LDPE to degradation, stemming from its stable carbon–carbon backbone and non-hydrolyzable covalent bonds, has led to persistent pollution in marine and terrestrial ecosystems [3]. Traditional waste management methods, such as incineration, recycling, landfilling, and bioremediation, have proven insufficient to address scaling of plastic pollution. These methods are resource intensive and fail to adequately mitigate the ecological and economic impacts of plastic waste [4,5]. Addressing this issue through effective plastic waste management can create economic opportunities, generating employment, stimulating local economies, and driving innovation in recycling technologies [6].

In recent years, the potential of biological degradation, particularly using fungal mycelium, has garnered attention as a viable approach to addressing plastic pollution [7]. Fungal mycelium, the vegetative network of fungi, exhibits a unique ability to produce extracellular enzymes such as laccases, manganese peroxidases, and lignin peroxidases, which can oxidize or hydrolyze complex polymers, including synthetic plastics [8]. These enzymes, combined with the fungi’s adaptability to diverse environmental conditions, make them interesting possibilities for the biological degradation of LDPE, LLDPE, and other plastics [9].

Studies have demonstrated that several fungal species, such as *Aspergillus flavus*, *Pleurotus ostreatus*, *Trametes versicolor*, and *Trichoderma harzianum*, exhibit potential for polyethylene (PE) degradation in laboratory and soil-simulated conditions [9,10,11,12]. Other fungi, such as *Phanerodontia chrysosporium*, *Phanerochaete chrysosporium*, *Ganoderma lucidum*, *Lentinula edodes*, and *P. ostreatus*, have shown enzymatic activity that modifies the chemical structure of LDPE and LLDPE, facilitating its mass loss and breakdown [7,13,14,15]. White-rot fungi, including *Agrocybe aegerita* and *G. lucidum*, are particularly noteworthy for their ligninolytic enzyme systems, which contribute significantly to the oxidative degradation of synthetic polymers [16]. Additional research highlights species like *Cladosporium* sp., *Collectotrichum fructicola*, *Diaporthe italiana*, *P. ostreatus*, *Stagonosporopsis citrulli*, and *Thyrostroma jaczewskii*, which alter LDPE’s surface morphology and chemical properties, further supporting the potential of fungal mycelium in plastic bioremediation [7,11,17,18].

Beyond LDPE and LLDPE, fungal species have demonstrated the capacity to degrade other plastics such as polyethylene terephthalate (PET), polystyrene (PS), polypropylene (PP), polyvinyl chloride (PVC), and polyurethane (PUR). For instance, *A. oryzae*, *Beauveria brongniartii*, *Fusarium oxysporum*, *Humicola insolens*, *Penicillium citrinum*, and *Pichia pastoris* have shown PET-degrading capabilities, while *Ceriporia* sp., *Cymatoderma dentriticum*, *P. ostreatus* and *Pycnoporus sanguineus* have been reported to degrade PS and PP, respectively [7,9]. These findings underscore the versatility of fungal mycelium in addressing a wide range of plastic waste, paving the way for its integration into sustainable waste management systems.

This study aims to explore the potential of fungal mycelium as a sustainable solution for LLDPE waste management by utilizing the growth, adaptability, and enzymatic capabilities of specific fungal species. This research proposes to fill important knowledge gaps in the biodegradation of plastics. The results will contribute to the development of eco-friendly, cost-effective waste management strategies, advancing a circular economy and minimizing the environmental footprint of synthetic polymers.

## 2. Materials and Methods

**Figure 1:**
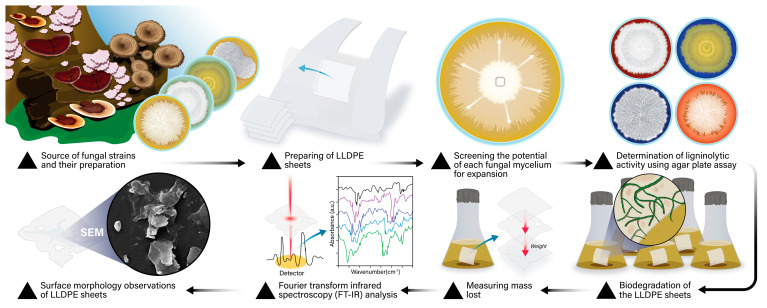
Visual overview of the testing process for breaking down LLDPE using fungal mycelium (part A).

The materials and methods section of the study covered several key steps, beginning with the sourcing and preparation of fungal strains, followed by the preparation of LLDPE sheets. The process included screening each fungal mycelium for expansion potential, assessing ligninolytic activity using an agar plate assay, and conducting biodegradation experiments on the LLDPE sheets. Subsequent analyses involved measuring mass loss, observing surface morphology, and performing Fourier Transform Infrared (FTIR) spectroscopic analysis, as illustrated in Figure 1.

### 2.1. Source of Fungal Strains and Their Preparation

The pure mycelia of ten fungal species were sourced from the culture collection of the Sustainable Development of Biological Resources Laboratory (*Lentinus squarrosulus* SDBR-CMU-WE001, *Panus conchatus* SDBR-CMU-WE021, *Phellinus linteus* SDBR-CMU-WE058, *Schizophyllum commune* SDBR-CMU-WE032, and *Trametes flavida* SDBR-CMU-AM011), Faculty of Science, Chiang Mai University, Thailand. Additional fungal species were obtained from the Thailand Bioresource Research Center (*Lentinus sajor-caju* TBRC6266) and the Thailand Mushroom Culture Collection (*Agrocybe cylindracea* TMCC-NO1, *Auricularia polytricha* TMCC-NO8, *Coprinus comatus* TMCC-NO1, and *Macrocybe crassa* TMCC-NO1). Prior to testing, all fungal species were cultured on potato dextrose agar (PDA; Conda, Madrid, Spain) and incubated at 30 °C for 7 days to provide optimal growth.

### 2.2. Preparation of LLDPE Sheets

LLDPE clear plastic tote bags (Chada, Saengroong Siam Plastic Company Limited, Nakhon Pathom, Thailand) with a thickness of 0.04 mm were selected and used for this study. The plastic bags were cut into 3 cm × 3 cm pieces and dried at 60 °C for 24 h. After drying, the sample sheets were weighed using a four-decimal precision scale, and their weights were recorded. Each piece was then placed separately in sterile Petri dishes for subsequent use.

The plastic pieces were soaked and sterilized in 75% ethanol for 3 h under a laminar flow clean bench. Following this, they were rinsed thoroughly 3 times with sterile deionized (DI) water and air-dried under sterile conditions in the laminar flow clean bench. Finally, the sheets were sterilized using ultraviolet (UV) light for 12 h to ensure aseptic conditions before being used in the experiment [17].

### 2.3. Screening the Potential of Each Fungal Mycelium for Expansion

The expansion of fungal mycelium for each species was evaluated on the surface of PDA plates, which were overlaid with sterilized cellophane disks. A 5 mm mycelial disk of each fungal species was placed at the center of separate PDA plates. The inoculated plates were incubated in complete darkness at 30 °C for 7 days. To assess mycelial growth, the diameters were measured daily, and growth rates were calculated by dividing the total mycelial diameter by the incubation period. After the incubation, the cellophane disks were carefully removed and dried at 80 °C for 24 h, as described by Aiduang et al. [19]. The density of the mycelium was determined qualitatively using the following scale adapted from Kalaw et al. [20], which included very thin (+), thin (++), thick (+++), and very thick (++++). This method allowed for both quantitative and qualitative assessment of fungal mycelium growth and density across different species.

### 2.4. Determination of Ligninolytic Activity Using Agar Plate Assay

Ten isolated fungal strains were screened for their lignin-degrading potential using different culture media. For the laccase (Lac) enzyme assay, the medium was prepared by supplementing potato dextrose agar (PDA) with 0.01% guaiacol and 0.01% Brilliant Blue R, following the method described by D’Souza et al. [21]. A modified basal medium, comprising KH_2_PO_4_ (1.0 g/L), (NH_2_)_2_CO (0.5 g/L), MgSO_4_·7H_2_O (0.5 g/L), CaCl_2_·2H_2_O (0.01 g/L), yeast extract (0.01 g/L), CuSO_4_·5H_2_O (0.001 g/L), Fe_2_(SO_4_)_3_ (0.001 g/L), MnSO_4_·H_2_O (0.001 g/L), 10 mM guaiacol (*v*/*v*), 10 mM H_2_O_2_, and 2.0% (*w*/*v*) agar, was used to determine lignin peroxidase (LiP) activity. The manganese peroxidase (MnP) assay was conducted using a Czapek Dox agar medium supplemented with 0.01% (*w*/*v*) phenol red, as previously described according to Kuwahara et al. [22] and Ali et al. [23]. All assay media were sterilized by autoclaving at 121 °C for 15 min. A 5 mm mycelial disk of each fungal strain was inoculated at the center of the assay plates. The inoculated plates were then incubated at 30 °C in darkness for 5 days. The clear decolorization or colorization zones observed in the Lac, LiP, and MnP activity assays were recorded to calculate the enzymatic index (EI). The EI was calculated using a modified method adapted from Lechuga et al. [24]. The amount of EI was determined based on the comparison of the average total diameter of the clear zone (CZ) with the average total colony diameter (CD). The calculation was performed using the following formula:

$$\mathrm{Enzymatic}\;\mathrm{Index}\;(\mathrm{EI})=\frac{\mathrm{CZ}\;-\;\mathrm{CD}\;}{\mathrm{CD}}$$

All experiments were performed in three replicates, and the results are presented as the mean value.

### 2.5. Biodegradation of the LLDPE Sheets

The selected three fungal species with high-performance growth and ligninolytic activity were evaluated for their biodegradation ability on prepared LLDPE sheets. Five mycelial plugs from each species (5 mm in diameter) were transferred into 100 mL of sterilized potato dextrose broth (PDB) medium, along with four sterilized LLDPE sheets. The cultures were incubated on a shaker at room temperature (25–29 °C) for 30 days to simulate conditions resembling natural environments. Control samples, consisting of sterilized LLDPE sheets without mycelial plugs, were included for comparison, following the method adapted from Gong et al. [17].

### 2.6. Measuring Mass Lost

The rate of biodegradation was assessed in this study by measuring the mass loss of LLDPE sheets. After 30 days of fungal mycelium inoculation, the LLDPE sheets were collected and washed overnight with 2% (*v*/*v*) sodium dodecyl sulfate (SDS) to remove any adhering mycelium and residues. Subsequently, the sheets were cleaned with 75% ethanol, rinsed thoroughly three times with distilled water, and dried in a hot-air oven. Once dried, the LLDPE sheets were weighed, and the percentage of weight loss was calculated using the following formula: weight loss percentage = [(initial weight − final weight)/initial weight] × 100 [17]. To further estimate the biodegradation rate, the weight loss was divided by the duration of the test period, providing an approximate value of weight reduction per day as an indicator of degradation efficiency.

### 2.7. Surface Morphology Observations of LLDPE Sheets

The surface morphology of the degraded LLDPE sheets from each treatment was analyzed using Scanning Electron Microscopy (SEM). Dried samples were cut into small rectangular pieces (approximately 5 × 5 mm) using a scalpel. Each sample was mounted onto a 10 mm^2^ stub adapter with 2 mm double-sided carbon tape. Subsequently, the samples were coated with gold for two minutes under high vacuum conditions. The prepared samples were examined and imaged using a scanning electron microscope (SEM JSM-IT300, JEOL, Tokyo, Japan) operating at an initial voltage of 15 kV. Imaging and analysis were conducted at the Science and Technology Service Center, Faculty of Science, Chiang Mai University, Thailand. Surface structures of the LLDPE sheets were compared across treatments based on the SEM images to identify morphological differences.

### 2.8. Fourier Transformed Infrared (FTIR) Spectroscopic Analysis

The chemical structure of the degraded LLDPE sheets from each treatment was analyzed using Fourier Transform Infrared Spectroscopy (FTIR) with the Nicolet 6700 FT-IR Spectrometer (Thermo Fisher Scientific, Waltham, MA, USA) for a detailed comparative study.

### 2.9. Statistical Analysis

The statistical analysis of all experiments was conducted using one-way analysis of variance (ANOVA) with the SPSS software program, version 17.0 (SPSS Inc., Chicago, IL, USA, for Windows). Duncan’s multiple range test was applied to identify any significant differences (*p* ≤ 0.05) between the mean values.

## 3. Results and Discussions

### 3.1. The Potential of Fungal Mycelium Expansion

The mycelial growth characteristics of various fungal species were evaluated based on their rate of expansion, dry weight estimation, and density level. Significant differences were observed across all parameters (Table 1), demonstrating distinct growth potential among the tested species. Among the species, *L. sajor-caju* (26.13 ± 0.91 mm/day) exhibited the highest mycelial expansion rate, followed by *S. commune* (20.53 ± 0.06 mm/day) and *T. flavida* (19.93 ± 0.06 mm/day), all of which showed significantly greater growth compared to other species. In contrast, *A. cylindracea* (5.74 ± 0.09 mm/day) and *C. comatus* (5.98 ± 0.91 mm/day) had the slowest mycelial expansion, with their growth rates being significantly lower than those of the fastest-growing species.

In terms of mycelial dry weight, *S. commune* had the highest biomass accumulation (276.87 ± 16.79 mg), significantly surpassing *T. flavida* (232.70 ± 9.17 mg) and *L. sajor-caju* (232.20 ± 15.98 mg), both of which also exhibited high biomass production. Conversely, *A. cylindracea* (7.07 ± 1.33 mg) and *C. comatus* (9.17 ± 2.58 mg) had the lowest mycelial dry weights, with values significantly lower than all other species.

**Figure 2:**
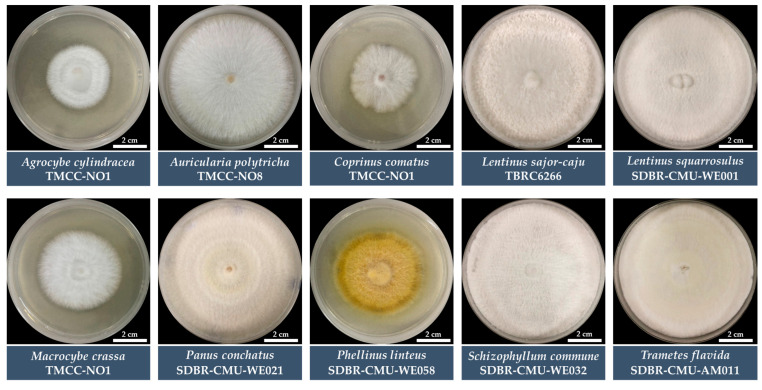
Growth characteristics of each fungal mycelium species for seven days on culture medium.

Mycelial density assessment revealed that species such as *L. sajor-caju*, *L. squarrosulus*, *S. commune*, *T. flavida*, *A. cylindracea*, and *M. crassa* developed very thick mycelium (++++), indicating strong and active growth. Meanwhile, *A. polytricha* and *P. linteus* displayed thick (+++) mycelium, while *C. comatus* and *P. conchatus* showed only thin density (++), suggesting a less compact mycelial network (Figure 2).

Overall, *L. sajor-caju*, *S. commune*, and *T. flavida* demonstrated superior mycelial growth potential in terms of expansion rate, biomass accumulation, and density, making them possible alternatives for applications requiring rapid and advantageous fungal biomass over time. In contrast, *A. cylindracea* and *C. comatus* exhibited significantly lower growth performance, indicating a slower and less dense mycelial development. These findings provide valuable insights into fungal cultivation efficiency and potential industrial applications.

The significant differences observed in fungal growth performance indicate that species with rapid expansion, high mycelial biomass accumulation, dense mycelial structures, along with species-specific variations in medium usage, which are critical for enzymatic activity and plastic degradation [9,25,26,27,28]. On the other hand, slow-growing fungal hyphae might have difficulty with competition, colonization, and contamination, which could impact their adaptability and effectiveness in environmental applications [6,29,30]. The extensive and thick mycelial networks of *S. commune*, *L. sajor-caju*, and *T. flavida* suggest a greater ability to penetrate and adopt plastic surfaces, enhancing biodegradation efficiency [25]. These findings demonstrate the importance of selecting highly potent fungal strains with strong growth characteristics for biotechnological applications in plastic waste management. Further studies should focus on optimizing growth conditions, enhancing enzyme expression, developing substrate accessibility, and assessing their long-term direct impact on LLDPE breakdown efficiency for sustainable plastic waste management and as feasible options in bioremediation.

### 3.2. Screening of Fungi for Ligninolytic Activity Using Agar Plate Assay

**Figure 3:**
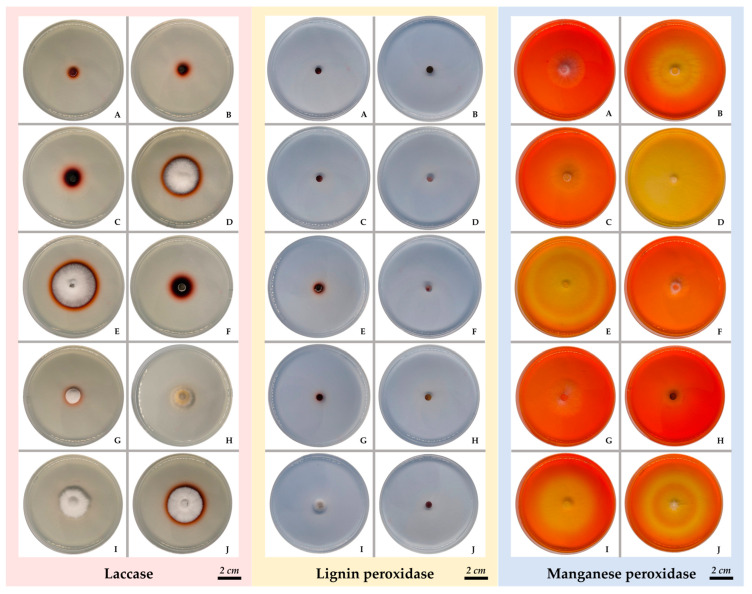
Ligninolytic activity (laccase, lignin peroxidase, and manganese peroxidase) of different fungal species using agar plate assay. (**A**) *A. cylindracea*; (**B**) *A. polytricha*; (**C**) *C. comatus*; (**D**) *L. sajor-caju*; (**E**) *L. squarrosulus*; (**F**) *M. crassa*; (**G**) *P. conchatus*; (**H**) *P. linteus*; (**I**) *S. commune*; (**J**) *T. flavida*. Scale bar: 2 cm.

Ligninolytic activity is the capacity of microorganisms, primarily fungi and bacteria, to breaks down lignin, a complex aromatic polymer found in plant cell walls. Interestingly, lignin-degrading enzymes such as Lac, LiP, and MnP have been demonstrated to contribute to the breakdown of synthetic polymers such as polyethylene (PE), high density polyethylene (HDPE), PP as well as LLDPE [7,31]. The preliminary evaluation of enzymatic activity was carried out using a plate assay on different agar media, with phenol red and Brilliant Blue R as indicators. Ten fungal species were subjected to this assay. Based on growth measurements, the fungal species showed varying mycelial diameters depending on the enzymatic agar used. Additionally, different strains exhibited distinct zone clearing, suggesting enzymatic activity and substrate degradation (Figure 3).

The EI of each fungal species in each enzyme assay is presented in Table 2. Among the fungal species tested for Lac activity, *C. comatus* exhibited the highest EI, with an average value of 4.97 ± 0.50. This was followed by *M. crassa*, *A. polytricha*, *P. conchatus*, and *A. cylindracea* with averages of 3.27 ± 0.42, 2.17 ± 0.89, 1.14 ± 0.04, and 1.04 ± 0.11, respectively. Additionally, other fungal species such as *L. sajor-caju*, *L. squarrosulus*, *S. commune*, and *T. flavida* also demonstrated potential Lac activity. These findings demonstrate the important Lac-producing capacity of these species, suggesting their application in bioremediation of phenolic compounds, pollutant detoxification, and industrial processes such as lignin degradation and waste management [18,32,33]. In contrast, the screening for LiP activity using a specific LiP screening medium, revealed that none of the tested fungal species showed significant enzyme production. This indicates that the conditions optimized for Lac synthesis did not promote LiP enzyme activity or that the species examined may naturally lack LiP production under these specific experimental conditions [32]. Furthermore, the investigation of MnP demonstrated positive results in four fungal species. *L. sajor-caju* showed the highest EI for MnP activity, with an average value of 0.36 ± 0.02, followed by *T. flavida*, *S. commune*, and *L. squarrosulus*, with average values of 0.35 ± 0.04, 0.28 ± 0.06, and 0.17 ± 0.02, respectively. These results suggest that several fungal species, particularly *L. sajor-caju*, *T. flavida*, and *S. commune*, exhibit strong ligninolytic potential due to their ability to produce both Lac and MnP. This makes them promising possibilities for future fungal biodegradation research, with potential applications in environmental remediation and industrial bioprocessing.

Overall, the observed differences in ligninolytic activity in this study are probably due to modifications in cultural and nutritional conditions. These findings are consistent with previous studies, which found that various fungus species produce varied amounts of enzyme activity depending on the conditions of cultivation of the fungus such as pH, temperature, carbon, and nitrogen sources [33,34]. For example, Viswanath et al. [33] reported that differences in ligninolytic enzyme activity among white-rot fungi were significantly influenced by the availability of inducers such as lignin-related compounds and specific growth conditions. Similarly, Wong [34] highlighted the critical role of environmental and nutritional factors in controlling ligninolytic enzyme expression across various fungal species. Such differences demonstrate the necessity of optimizing growing conditions to improve enzyme efficiency for industrial and biotechnological applications.

### 3.3. Weight Loss Measurement

**Figure 4:**
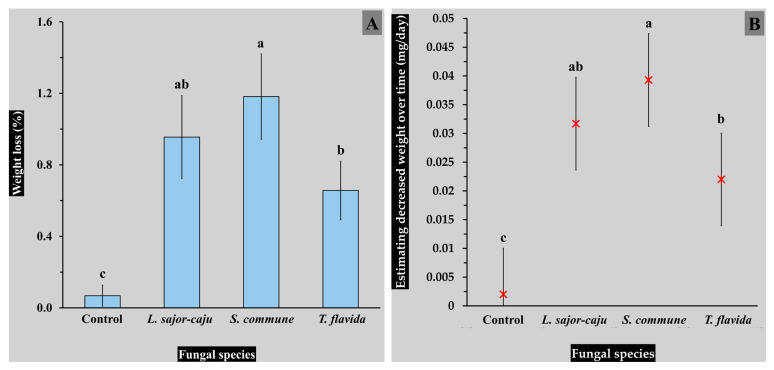
The percentage weight loss of LLDPE sheets after 30 days using the biodegradation process with fungal mycelium (**A**) and the estimated weight loss over time (**B**). The data are presented as means, with error bars representing the standard deviation for each point. Statistical significance was analyzed using Duncan’s multiple range test, where distinct letters (a–c) indicate significant differences (*p* ≤ 0.05).

The results of the study demonstrated significant differences in the percentage weight loss of LLDPE sheets after 30 days of fungal mycelium-mediated biodegradation (Figure 4), demonstrating the role of fungal species play in stimulating plastic degradation. Among the tested species, *S. commune* achieved the highest weight loss (1.182 ± 0.239%), significantly above the control (0.068 ± 0.059%) and other fungal species. This indicates that *S. commune* possesses strong enzymatic and metabolic capabilities to break down LLDPE, likely due to its production of extracellular enzymes like laccases and manganese peroxidase [9].

*Lentinus sajor-caju* also exhibited notable biodegradation activity, with a weight loss of 0.955 ± 0.232%, statistically comparable to *S. commune* but significantly higher than *T. flavida* (0.657 ± 0.164%) and the control. Meanwhile, *T. flavida* AM011 showed moderate biodegradation potential, achieving a weight loss significantly greater than the control, yet being less effective than *S. commune* and *L. sajor-caju*. The control treatment, without fungal intervention, exhibited minimal weight loss, confirming the necessity of fungal activity for effective degradation of LLDPE sheets.

Based on the estimated weight loss per day in the degradation of LLDPE, all fungal strains tested showed significantly higher degradation rates compared to the control (0.002 mg/day). Among the fungi, *S. commune* exhibited the most effective degradation, with a rate of 0.0393 mg/day, followed by *L. sajor-caju* (0.0317 mg/day) and *T. flavida* (0.022 mg/day). The results indicate that S. commune WE032 stimulated LLDPE degradation nearly 20 times faster than the control, making it potentially promising option for plastic degradation. This significant difference demonstrates the potential for applying fungal biotechnology in easier and more sustainable plastic waste management solutions.

Statistical analysis indicated clear differences between the treatments, with significant groupings identified. These findings demonstrate the critical importance of fungal species selection in optimizing biodegradation processes. The superior performance of *S. commune* suggests it as a good option for sustainable plastic waste management because of its efficient degradation capabilities and possibility to decrease processing times. Further research into the enzymatic mechanisms and environmental conditions supporting its activity could enhance its practical application in large-scale plastic waste treatment.

When compared to previous studies on fungal biodegradation of plastics, which reported percentage weight losses ranging from 0.2 to 50% over 15–240 days, this study aligns with findings that highlight variability based on fungal species, conditions, durations, and evaluation methods. Fungal species such as *A. flavus*, *A. tubingenesis*, *Candida antarctica*, *Cladosporium* sp., *P. chrysosporium*, *P. citrinum, T. harzianum*, *G. lucidum*, *P. ostreatus*, and *T. versicolor* have been commonly studied [7,9,17,18]. For LDPE and LLDPE biodegradation, weight losses of 0.20–46.34% were observed across 30–150 days in various studies [7,11,15,17,18,35]. In this study, the percentage weight loss of LLDPE sheets ranged from 0.657 to 1.182% after 30 days (with an effective degradation rate of 0.022–0.0393 mg/day), demonstrating positive outcomes when compared with many previous investigations [17,36]. This suggests the potential of fungal mycelium as an alternative approach for managing plastic waste, particularly in regions with high levels of LLDPE and LDPE pollution.

### 3.4. Surface Morphology of LLDPE Sheets

**Figure 5:**
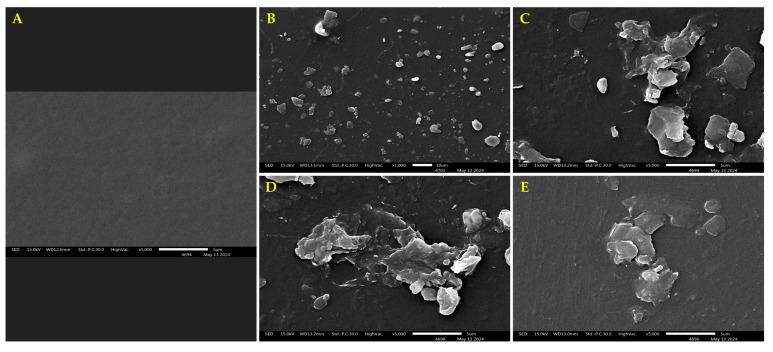
The degraded LLDPE plastic sheets displayed apparent surface erosion caused by fungal mycelium in each tested species, compared to the untreated control samples. (**A**) Surface morphology of LLDPE control sheets; (**B**) overall visible erosion on a degraded plastic sheet; (**C**) LLDPE plastic sheets degraded by *L. sajor-caju*; (**D**) LLDPE plastic sheets degraded by *S. commune*; and (**E**) LLDPE plastic sheets degraded by *T. flavida*.

The investigation of the surface morphology of LLDPE plastic sheets subjected to biodegradation by fungal mycelium revealed notable differences compared to the untreated control samples. The degraded plastic sheets exhibited visible surface erosion, indicating the mycelium’s ability to alter the material’s structure. This observation aligns with the weight loss measurements obtained during the study. Specifically, the surface erosion was most pronounced in plastic sheets treated with *S. commune* and *L. sajor-caju* mycelium, as shown in Figure 5. These sheets displayed extensive erosion marks, consistent with their significant weight loss. In contrast, the plastic sheets treated with *T. flavida* mycelium exhibited only slight surface erosion, indicating a comparatively lower biodegradation efficiency. These findings support the weight loss data and confirm the biodegradation potential of specific fungal strains.

These findings underscore the potential of fungal mycelium as an effective biodegradation agent for LLDPE, commonly found that the aging process resulted in changes in structural properties (insertion of functional groups), as well as morphological (appearance of micro-cracks and increased roughness) [37]. The degree of surface erosion observed correlates with the weight loss results, reinforcing the role of fungal enzymatic activity in degrading plastic polymers. *S. commune* and *L. sajor-caju* demonstrated superior performance, likely due to their robust enzymatic systems, such as laccases and peroxidases, which can effectively break down the carbon-carbon bonds in LLDPE. The lower degradation efficiency of *T. flavida* may stem from less active enzymatic pathways or reduced adaptability to the experimental conditions. These results highlight the variability in biodegradation potential among fungal species, emphasizing the importance of selecting and optimizing fungal strains for specific applications.

### 3.5. FTIR Analysis

**Figure 6:**
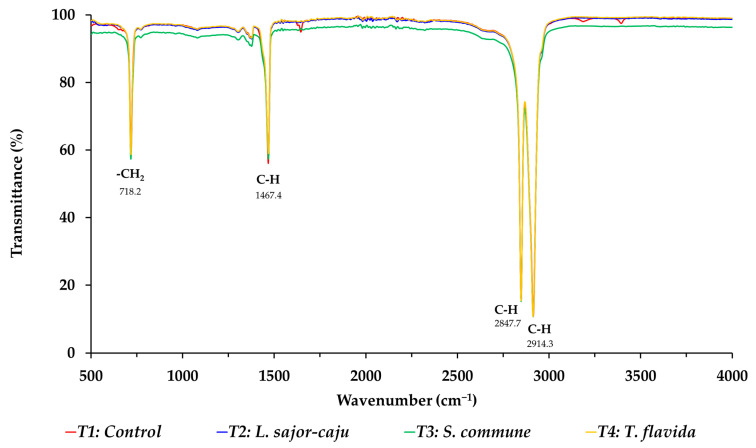
FTIR spectrum of LLDPE sheets after 30 days of biodegradation by fungal mycelium.

The FTIR spectra of the primary and biodegraded LLDPE sheets are presented in Figure 6. It was revealed that the FTIR spectroscopy of the primary (T1) LLDPE sheet showed absorption bands at 2914.3 cm^−1^ for –CH asymmetric stretching, 2847.7 cm^−1^ for –CH symmetric stretching, and 1467.4 cm^−1^ for −CH bending deformation. For the LLDPE sheet that was biodegraded by *S. commune* (T3) for 30 days, a reduction in most of the FTIR peak intensities was observed, which was caused by changes in the polymer structure. The peaks observed in Figure 6 at 1467.4–1468.2 cm^−1^ displayed slight changes. Additionally, the decreased intensities at 2914.3 cm^−1^ (asymmetric CH_2_ stretch) and 2847.7 cm^−1^ (symmetric CH_2_ stretch) indicate degradation of the polymer backbone. A similar result was highlighted by Sangale et al. [38] and Jung et al. [39] who observed a decrease in peak intensity in the 2800–3000 cm^−1^ region for microbially degraded polyethylene.

Previous studies have shown that microbial degradation leads to a reduction in the intensity of these bands due to the breakdown of polymer chains [17,31]. LLDPE structural changes due to microbial activity confirmed the development of new functional groups like hydroxyl and carbonyl, which researchers also reported in the study of Sathiyabama et al. [36]. Microbial enzymes facilitate oxidative degradation through spectral changes, which include new peak appearance and existing peak shifts. Water-absorbing oxygen-attracted functional group formation in the polymer material improves hydrophilic conditions and increases sensitivity to breakdown [31,40]. The FTIR analysis confirms that the investigated fungal species used in this study lead to substantial structural modifications in LLDPE, which are shown through observed transitions in characteristic absorption peaks. The new functional group formation during microbial breakdown indicates potential applications of these strains for LLDPE biodegradation, while the backbone aliphatic structure shows signs of breakdown according to these findings.

### 3.6. Cost Effectiveness and Economic Impact in Management

The cost-effectiveness and economic impacts of plastic waste treatment methods vary significantly across different approaches (Table 3). Incineration, though capable of energy recovery, comes with high initial setup and operational costs ($36–578.5 per ton). It offers heat and electricity production, reducing emissions in the energy sector, but it has substantial carbon emissions and secondary pollution concerns if not properly managed. On the other hand, landfilling is low in cost ($6.02–19 per ton) but poses environmental risks such as water and soil pollution, with slow decomposition of plastics and difficult long-term management.

Although recycling is environmentally beneficial and aligned with circular economy goals, it has high operational costs ($240–1800 per ton) and is applicable only to specific plastic types, requiring complex technology for processing. It has moderate carbon emissions, but economic benefits include the production of new plastics and valuable byproducts.

Fungal biodegradation offers a promising low-cost solution for plastic waste management. Its operational costs are dependent on feedstock and local availability, and it produces significantly lower carbon emissions compared to traditional methods. Fungal processes are simpler and can provide economic benefits such as the creation of job opportunities, green technology innovation, and enhanced local economies through bio-remediation. It aligns with sustainable waste management practices and has the potential to generate value-added products while mitigating harmful emissions from other plastic waste treatments.

Overall, while incineration and recycling offer certain economic benefits, they come with high costs and environmental impacts. Fungal biodegradation, though requiring significant initial investment in biotechnology research and development, presents a more sustainable and cost-effective alternative with lower carbon emissions, contributing to both environmental and economic advantages.

### 3.7. Future Perspectives and Further Activities

Fungal bioremediation offers a sustainable and cost-effective solution for environmental preservation, but scaling up and practical application present key challenges [8]. These include the selection of effective fungal species capable of degrading specific compounds and adapting to varying environmental conditions. Contaminant complexity is another challenge, as fungi may have troubles with mixed pollutants or substances toxic to their growth. Transitioning from lab-scale studies to large-scale applications requires process optimization, including parameters like essential nutrients, moisture, temperature, and oxygen availability. Additionally, site-specific factors such as pH, temperature, and soil composition must be carefully evaluated to ensure feasibility and adaptation for successful fungal bioremediation [6].

**Figure 7:**
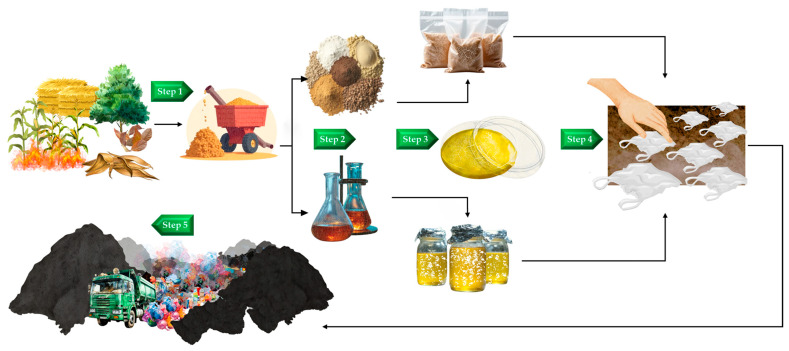
A pathway for transferring fungal mycelium-based bioremediation from the laboratory to the field.

To address these challenges, this study plans to develop fungal mycelium bio-prototypes as bioremediation substances (Figure 7), with focusing on utilizing biomass residues from agriculture and forestry sectors, such as corn stalks, rice straw, sugarcane leaves, and forest leaf litter. These materials, which cause burning and PM 2.5 problems, will be ground into small pieces and used as a medium to cultivate S. commune. The biomass will be converted into two types of products, including solid mushroom spawns for spreading in landfill areas and semi-synthetic culture media for the application of spray. Both prototypes will be invested through LLDPE deterioration tests in simulated environments (soil burial) before being applied to real landfill conditions.

The research team expects that, if successful, this study will provide a cost-effective and environmentally friendly approach to plastic waste management. Beyond addressing plastic waste, this method has the potential to positively impact the environment by utilizing abundant, low-cost biological resources more effectively. Additionally, it could help reduce burning and PM 2.5 pollution while generating job opportunities in regions that benefit from its implementation.

## 4. Conclusions

The study demonstrates the potential of fungal mycelium, particularly *S. commune* and *L. sajor-caju*, as effective facilitators for the biodegradation of LLDPE. The findings demonstrate that these fungal species exhibit superior growth characteristics, high enzymatic activity, and significant weight loss of LLDPE sheets, suggesting their capability to degrade plastic polymers efficiently. The observed surface erosion and changes in morphology further support their biodegradative potential, displaying the critical role of enzymatic processes in the breakdown of synthetic polymers. Moreover, FTIR analysis confirmed substantial structural modifications in LLDPE after fungal biodegradation. Sheets biodegraded by *S. commune* exhibited reduced intensity in characteristic peaks and the emergence of new functional groups, such as hydroxyl and carbonyl groups, indicating polymer chain scission and oxidative degradation. These chemical changes suggest enhanced hydrophilicity and greater biodegradability, supporting the possibility that fungi impact the backbone of polymers. Beyond its environmental benefits, fungal biodegradation presents a viable and cost-effective alternative to conventional plastic waste management methods such as incineration and landfilling. With significantly lower operational costs and reduced carbon emissions, fungal remediation aligns with circular economy principles while offering opportunities for sustainable development, including green technology innovation and work creation. However, challenges remain in scaling up this approach for industrial applications, particularly in optimizing fungal growth conditions, enhancing enzymatic activity, and addressing site-specific environmental factors. Future research should focus on improving fungal adaptability to diverse plastic waste environments, optimizing enzyme production, and integrating biomass residues as a sustainable growth medium. By utilizing agricultural and forestry byproducts, fungal-based bioremediation can contribute not only to plastic waste reduction but also to mitigating air pollution and promoting a circular bioeconomy. The positive findings of this study provide the possibility for further exploration of fungal biotechnology in sustainable waste management, potentially transforming plastic pollution into an opportunity for environmental and economic sustainability.

## Figures and Tables

**Table 1:** Potential of fungal mycelium growth in each species.

Fungal Species	Rate of Mycelial Expansion (mm/Day)	Mycelial Dry Weight Estimation (mg)	Level of Mycelial Density
*A. cylindracea* TMCC-NO1	5.74 ± 0.09 ^h^	7.07 ± 1.33 ^f^	++++
*A. polytricha* TMCC-NO8	11.82 ± 0.09 ^e^	72.87 ± 14.63 ^d^	+++
*C. comatus* TMCC-NO1	5.98 ± 0.91 ^gh^	9.17 ± 2.58 ^ef^	++
*L. sajor-caju* TBRC6266	26.13 ± 0.91 ^a^	232.20 ± 15.98 ^b^	++++
*L. squarrosulus* SDBR-CMU-WE001	19.50 ± 0.69 ^c^	166.60 ± 16.57 ^c^	++++
*M. crassa* TMCC-NO1	6.70 ± 0.71 ^g^	28.70 ± 3.76 ^e^	++++
*P. conchatus* SDBR-CMU-WE021	12.89 ± 0.28 ^d^	148.60 ± 9.37 ^c^	++
*P. linteus* SDBR-CMU-WE058	9.03 ± 0.19 ^f^	25.73 ± 6.31 ^ef^	+++
*S. commune* SDBR-CMU-WE032	20.53 ± 0.06 ^b^	276.87 ± 16.79 ^a^	++++
*T. flavida* SDBR-CMU-AM011	19.93 ± 0.06 ^bc^	232.70 ± 9.17 ^b^	++++

**Note:** Mean ± SD values in the same column that have different lowercase superscripts are significantly different (*p* ≤ 0.05). Mycelium density was qualitatively assessed using a four-level scale: thin (++), thick (+++), and very thick (++++).

**Table 2:** Enzymatic index of fungal species in each enzyme assay.

Fungal Species	Enzymatic Index (EI)		
	Laccase	Lignin Peroxidase	Manganese Peroxidase
*A. cylindracea* TMCC-NO1	1.04 ± 0.11 ^d^	0.00 ± 0.00	0.00 ± 0.00 ^d^
*A. polytricha* TMCC-NO8	2.17 ± 0.89 ^c^	0.00 ± 0.00	0.00 ± 0.00 ^d^
*C. comatus* TMCC-NO1	4.97 ± 0.50 ^a^	0.00 ± 0.00	0.00 ± 0.00 ^d^
*L. sajor-caju* TBRC6266	0.33 ± 0.02 ^e^	0.00 ± 0.00	0.36 ± 0.02 ^a^
*L. squarrosulus* SDBR-CMU-WE001	0.29 ± 0.01 ^e^	0.00 ± 0.00	0.17 ± 0.02 ^c^
*M. crassa* TMCC-NO1	3.27 ± 0.42 ^b^	0.00 ± 0.00	0.00 ± 0.00 ^d^
*P. conchatus* SDBR-CMU-WE021	1.14 ± 0.04 ^d^	0.00 ± 0.00	0.00 ± 0.00 ^d^
*P. linteus* SDBR-CMU-WE058	0.00 ± 0.00 ^e^	0.00 ± 0.00	0.00 ± 0.00 ^d^
*S. commune* SDBR-CMU-WE032	0.40 ± 0.03 ^e^	0.00 ± 0.00	0.28 ± 0.06 ^b^
*T. flavida* SDBR-CMU-AM011	0.35 ± 0.10 ^e^	0.00 ± 0.00	0.35 ± 0.04 ^a^

Note: Mean ± SD values in the same column that have different lowercase superscripts are significantly different (*p* ≤ 0.05).

**Table 3:** Comparison of cost effectiveness and economic impacts of plastic waste treatment methods.

Cost-Efficiency and Economic Impact	Waste Management Methods			
	Incineration	Landfilling	Recycling	Fungi
**Initial setup cost**	• High capital investments	• Low costs for the general filling pit, reclamation, orogeny; higher cost for sanitary landfill	• High implementation costs in management system • High energy cost • High oil cost • Affordability	• High initial costs associated with funding biotechnology facilities or research and development
**Operational cost**	• High cost ($36–578.5/ton)	• Low cost ($6.02−19/ton)	• High cost ($240–1800/ton)	• Low-cost solution for managing synthetic waste, fungal biodegradation requires specific growth media to produce enzymes, with costs depending on feedstock and local availability
**Environmental impact**	• If the flue gas is improperly treated, the secondary pollution will be very serious, whilst it can be controlled by advanced incineration or purification technology	• Pollutes the water and soil, which may affect wildlife and contribute to spreading harmful diseases • The secondary pollution of landfill leachate is serious and difficult to control	• High volumes of waste available • Environmental awareness	• Contribute to a bioremediation process more effective
**Carbon emissions**	• High carbon emissions as a result from burning plastic waste	• Moderate emissions in the biodegradation process	• Low to moderate emissions across the turning process	• Lower carbon emissions compared to traditional methods
**Expected economic benefits**	• Heat and electricity production leading to fewer emissions in the regular energy production sector • No sorting required, hence less expensive collection cost for solid wastes • Energy recovery	• Managing large volumes of plastic waste at low cost • Reducing transportation distances • Eliminating energy consumption.	• Avoidance of CO_2_ that otherwise would be emitted during incineration • Processing of materials which cannot otherwise, be recycled to value-add product • Production of new plastics and products which constitute good energy sources	• Utilizes living organisms, like fungi and bacteria, as a cost-effective and ecologically friendly method that could offer open business opportunities • A suitable waste disposal system and bio-remediation can improve local economies, provide employment, and support green technology innovation
**Technical process**	• Complicated	• Simple	• Complicated	• Simple
**Limits**	• High volumes to be processed per plant	• Slowly decomposes in landfill settings, requiring a large amount of area because of the rising rate from plastic waste disposal • Maintaining landfills in an ecologically friendly state for a long period is difficult	• Only applicable for selected plastic types collected in large volumes • Complex technology. • High volumes to be processed per plant	• Decomposition could require a long time • Its rate of decomposition mainly depends on pH, temperature, humidity, sunlight, UV radiation, and soil composition. • Different fungal species might exhibit varied specific substrates
**Main causes**	• Lack of space for landfilling • High demand and tariffs for electricity and hot water • Policy promotes incineration	• Lack of infrastructure • Poor administration • Insufficient rules	• Carbon credits • Policy promotes recycling	• Fungal biological potential (enzyme production) • Complies with the circular economy and sustainable waste management practices
**References**	[41,42,43,44,45,46]	[4,21,42,43,44,45,46,47,48]	[42,44,45,48]	[6,9,46,49,50]

## Data Availability

Data are contained within the article.
